# The structure of *Escherichia coli* ExoIX—implications for DNA binding and catalysis in flap endonucleases

**DOI:** 10.1093/nar/gkt591

**Published:** 2013-07-02

**Authors:** Christopher S. Anstey-Gilbert, Glyn R. Hemsworth, Claudia S. Flemming, Michael R. G. Hodskinson, Jing Zhang, Svetlana E. Sedelnikova, Timothy J. Stillman, Jon R. Sayers, Peter J. Artymiuk

**Affiliations:** ^1^Department of Molecular Biology and Biotechnology, Krebs Institute, University of Sheffield, Firth Court, Western Bank, Sheffield S10 2TN, UK and ^2^Department of Infection & Immunity, Krebs Institute, University of Sheffield Medical School, Beech Hill Road, Sheffield S10 2RX, UK

## Abstract

*Escherichia coli* Exonuclease IX (ExoIX), encoded by the *xni* gene, was the first identified member of a novel subfamily of ubiquitous flap endonucleases (FENs), which possess only one of the two catalytic metal-binding sites characteristic of other FENs. We have solved the first structure of one of these enzymes, that of ExoIX itself, at high resolution in DNA-bound and DNA-free forms. In the enzyme–DNA cocrystal, the single catalytic site binds two magnesium ions. The structures also reveal a binding site in the C-terminal domain where a potassium ion is directly coordinated by five main chain carbonyl groups, and we show this site is essential for DNA binding. This site resembles structurally and functionally the potassium sites in the human FEN1 and exonuclease 1 enzymes. Fluorescence anisotropy measurements and the crystal structures of the ExoIX:DNA complexes show that this potassium ion interacts directly with a phosphate diester in the substrate DNA.

## INTRODUCTION

All cells require 5′-nuclease or flap endonuclease (FEN) activity for DNA replication and repair processes (1). For example, FEN activity is involved in the removal of RNA from Okazaki fragments, which are formed on the lagging-strand during semi-discontinuous DNA replication. Okazaki fragment synthesis requires a short RNA oligonucleotide primer. These can form a ‘flap’ of single-stranded nucleic acid when the DNA polymerase extending from an upstream primer carries out strand displacement synthesis. The unstable RNA must be removed to maintain genomic integrity, a process accomplished by a combination of ribonuclease H (RNAse H) and 5′ nuclease activities (2). Thus, an exonuclease is required to remove fully base-paired ribonucleotides at the 5′ end of the fragment, whereas an endonuclease is required should the 5′ end become displaced from the template strand and form a flap structure. FENs, Mg^2+^-dependant metalloenzymes that accomplish both these activities, comprise a group of proteins that are ubiquitously represented in all three superkingdoms of life (1). Structural studies have revealed a common FEN architecture, consisting of a central β-sheet flanked by α-helical domains [e.g. (3–10)]. The β-sheet carries many of the conserved acidic residues, which cluster at the centre of the enzyme forming the Cat1 and Cat2 metal-binding sites responsible for the catalytic activity of these enzymes (Supplementary Figure S1A) (11). These sites coordinate two divalent metal ions, which in prokaryotes have been observed to be separated by a distance of ∼8 Å. In *Homo sapiens* FEN homologue (hFEN1), Shen and coworkers suggested that one of the metal-binding sites (Cat1) is required for activating nucleophilic attack on the scissile phosphate diester bond, whereas the second site (Cat2) may stabilize the enzyme-substrate complex (12). In the bacteriophage T5 homologue, Cat1 is both essential and sufficient for structure-specific hydrolysis (flap cleavage) while both sites are required for 5′–3′ exonucleolytic activity (13). Kinetic analyses of a mutant engineered so as to lack two conserved aspartate groups in Cat2 suggests that this site is involved in substrate binding rather than chemical catalysis (14).

Steitz and coworkers (6) suggested that a two-metal-ion mechanism may operate in FENs, similar to that seen in the Klenow 3′–5′ proof-reading exonuclease (15) based on their structure of TaqPol (6). However, the deposited structure (1TAQ.pdb) contains only one bound divalent metal ion (Zn^2+^) and no DNA. This mechanism for phosphoryl transfer has been the subject of some controversy, given the large distance observed between the metals in prokaryotic FEN structures (16) but has received strong support from the hFEN1 and human exonuclease 1 (hExo1) structures. Both reveal pairs of metal ions in their Cat1 sites (Sm^3+^ in hFEN1 and Ba^2+^ and Mn^2+^ in hExo1) separated by ∼4 Å and coordinating a DNA phosphate group (9,10). However, it should be pointed out that none of these structures contained the biologically relevant metal cofactor ion magnesium; indeed, to date there are no published structures of a FEN enzyme with DNA and the *in vivo* cofactor Mg^2+^.

Above the active site is a region that plays important roles in substrate recognition and binding (17). Surprisingly, this region is highly variable structurally: it is an ordered helical arch in bacteriophage T5 exonuclease (T5FEN) but disordered in other FEN homologue structures such as bacteriophage T4 RNAse H and *Thermus aquaticus* (Taq) polymerase (6,7). The presence of the helical arch in T5FEN led to the proposal of a model of substrate binding in which the ssDNA 5′ arm of the flap threads through the hole formed by the arch (3). The structure of a T4 RNAse H mutant in complex with a pseudo-Y DNA molecule appears to support such a threading model, although the loop above the active site was not directly observed in the structure (4).

A helix-3-turn-helix (H3TH) or similar motif present in these proteins is also implicated in DNA binding (5). This is similar to the well-known helix-hairpin-helix (HhH) motif (18), except that the hairpin region is significantly extended into a loop carrying two aspartate Cat2 ligands (19). The H3TH motif is present in all FEN structures determined to date, but the length of the turn region varies, and in hFEN1 and hExo1, it is an H2TH motif (9,10). The similarity of this motif to the HhH motif extends to the sequence level. The glycine-hydrophobic-glycine (GhG) sequence motif, which mediates protein:DNA interactions through the backbone amide groups of the HhH motif (20), is also present in many FENs, suggesting that the H3TH motif may interact with DNA in a similar manner (5). The first FEN structure with DNA bound in this region of the protein was that of T4 RNAse H in complex with a pseudo–Y substrate (4), but T4 RNAse H lacks the GhG sequence motif. However, the structures of human FEN1 (9) and hExo1 (10) have revealed these interactions and additionally show the presence of a K^+^ ion, which further stabilizes the interaction.

In prokaryotes, the essential FEN reaction can be performed by the N-terminal 5′-3′ exonuclease domain present on DNA polymerase I. For example, in bacteria such as *Streptococcus pneumoniae* (21) and *Synechococcus elongatus* (22), the FEN domain of DNA polymerase I (PolI) is essential, as they do not encode any other FEN genes. In contrast, archaea (5), eukaryotes (23), bacteriophages and some viruses (24,25) encode a separate FEN enzyme but lack FEN domains on their DNA polymerases.

Intriguingly, however, it is now clear that many eubacteria possess a second FEN-encoding gene (*xni*) in addition to their PolI FEN domain (26). In many of these, including *Staphylococcus aureus* and *Bacillus subtilis*, the second FEN possesses both the Cat1 and the Cat2 metal-binding sites discussed earlier in the text. However, a subset of genera, for example *Erwinia*, *Escherichia*, *Klebsiella*, *Salmonella*, *Vibrio* and *Yersinia*, encode a second FEN (designated ExoIX), which lacks the three aspartate residues that make up the Cat2 site (22,26) (Supplementary Figure S1B). Fukushima *et al.*(22) have clearly demonstrated that either ExoIX or the DNA pol I FEN-domain is essential for cell viability, as in *E. coli* and *B. subtilis*, null mutants in either the FEN-encoding domain of *polA* or *xni* were viable, whereas double-mutants were not (22).

Here, we present the crystal structures of wild-type *E. coli* ExoIX determined both in the presence and absence of potassium and of divalent metal ions, and with and without bound DNA. We show that in ExoIX, the Cat2 site is abolished, but a pair of oxo-bridged Mg^2+^ ions is observed in the Cat1 site, lending further support to the two-metal-ion hypothesis in this important class of FENs. Given the high degree of sequence and structure conservation at the Cat1 site in all bacterial FENs, our results suggest that other prokaryotic FENs may bind two Mg^2+^ ions in their Cat1 sites, and that the two-metal-ion mechanism is conserved across the phyla. Fluorescence anisotropy (FA) studies and co-crystallization with DNA oligomers show that ExoIX, like its eukaryotic counterparts, exhibits potassium-dependent DNA-binding via a K^+^ site situated in its H3TH motif region.

## MATERIALS AND METHODS

### ExoIX expression and purification

The protein was overexpressed and purified as described by Hodskinson *et al.*(27). Subsequently, a potassium-free purification protocol was devised whereby the cells were lysed in 50 mM Tris–HCl (pH 8.0), 0.5 M NaCl, 1 mM DTT using sonication. Cell debris was removed by centrifugation at 43 700*g* for 20 min. The supernatant was then diluted 5-fold with 50 mM Tris–HCl (pH 8.0), 1 mM DTT and loaded onto a 20 ml heparin-Sepharose (GE Healthcare) column. The protein was eluted with a 0.1–0.5 M NaCl gradient over 150 ml. Fractions containing ExoIX were combined and concentrated to a volume of 1–1.5 ml. The sample was then diluted 10-fold with 50 mM Tris–HCl (pH 8.5) and applied to a 6 ml Resource-Q column through a 0.2 µm filter. The protein was then eluted by applying a salt gradient from 0 to 150 mM, NaCl in 60 ml and then 150 to 1000 mM in 30 ml, and fractions containing ExoIX were again pooled and concentrated. The protein was then applied to a HiLoad Superdex 200 size exclusion column equilibrated with 10 mM 2-(N-morpholino)ethanesulfonic acid (MES) (pH 6.5), 250 mM NaCl. Fractions containing ExoIX were combined and concentrated to between 15 and 25 mg/ml for crystallization.

### ExoIX crystallization

Crystal screens were undertaken using Hampton Research Crystal Screens 1 and 2, and the PEG/ion screen. Initial hits were optimized to produce crystals of diffraction quality. The first crystals were grown from 0.2 M sodium thiocyanate, 20% PEG 3350, 0.1 M MES at pH 6.5 at 17°C and belonged to the space group P1 with cell dimensions of a = 43.4 Å, b = 56.5 Å, c = 60.2 Å, α = 110.6°, β = 95.4°, γ = 95.1°, and diffracted to 2.0 Å resolution. Subsequently, a second crystal form was grown in space group P2_1_ under similar conditions but in the presence of ZnCl_2_ with cell dimensions a = 53.5 Å, b = 38.3 Å, c = 59.7 Å, α, γ = 90.0°, β = 108.0°. Crystals of the K^+^-free protein were grown in 0.2 M sodium acetate trihydrate, 20% PEG 3350 and were in the space group C2 with cell dimensions a = 128.5 Å, b = 37.4 Å, c = 66.7 Å, α, γ = 90.0°, β = 117.7°.

### ExoIX:DNA complex crystallization

Pure ExoIX and DNA were mixed in a 1:1 molar ratio in 10 mM MES (pH 6.0), 150 mM KCl, as this resulted in good DNA binding by the protein during FA measurements. Initial crystallization conditions were identified in the NeXtal PEGs screen and refined to 15% (w/v) PEG-3350 in the presence of 0.2 M magnesium acetate at 7°C.

### Crystallographic data collection and processing

Crystals were cryoprotected (except for the K^+^-free set, which was collected at room temperature mounted in a capillary) and exposed to X-rays. For an in-house data collection, a MAR345 image plate was used mounted on a Micromax 007 with a copper rotating anode. High-resolution data were collected at station PX14.1 of the SRS Daresbury Laboratory on a Quantum Q4 CCD detector or later at beamlines I02 or I03 on a ADSC Q315 CCD detector at the Diamond synchrotron, as detailed in Supplementary Table S1. Data processing was performed using MOSFLM in house (28), HKL2000 (29) at the Daresbury SRS and the CCP4 suite of programs (30). Procedures and statistics are given in Supplementary Table S1.

### Structure solution

The initial structure was solved by multiple isomorphous replacement in the P1 crystal form (Supplementary Table S1). Two derivatives were used: mercury (two sites) and gold (four sites), to 2.5 and 2.8 Å, respectively, and phases were then improved by solvent flattening and phase extension to 2.0 Å resolution (30). The structure was built and refined (see Supplementary Table S1 for details) and subsequently used to solve the second native structure in space group P2_1_ by molecular replacement using PHASER (31). The K^+^-free structure in space group C2, and the ExoIX:DNA complex structures in space group P2_1_2_1_2 were solved by molecular replacement with PHASER (31) using the P2_1_ structure as a search model. Model building was performed using TURBO (32) and COOT (33). REFMAC5 was used for structural refinement (34). Molecular diagrams were generated using Pymol (35) and CCP4MG (36). To calculate simulated annealing omit maps, the relevant atoms were removed from the model and three cycles of refinement were performed in phenix.refine (37) including Cartesian-simulated annealing after the second and last macro-cycles.

### FA measurements

All oligonucleotides used during this work were purchased HPLC purified from Eurofins MWG Operon. The substrates shown in Supplementary Figure S2 were formed by annealing the oligos with one another by incubation at room temperature for 10 min. Annealing was confirmed by analysing the oligos on a 10% native polyacrylamide gel to observe a shift in the migration of the DNA relative to the unannealed oligos by UV shadowing.

FA measurements were performed at 25°C in triplicate with excitation and emission wavelengths of 495 and 515 nm, respectively, in a Carey Eclipse Fluorescence Spectrophotometer supplied with a Varian Manual Polarizer. The excitation and emission slit widths were set at 5 nm, and the instrument has excitation and emission monochromators with built-in filters on the monochromators to minimize light scattering and exclude stray light. All experiments were performed in 10 mM MES (pH 6.0) with the annealed oligo at 15 nM. The Bradford Assay was used to measure the ExoIX concentration before the experiment. The equation used to fit the data in Figure 2A–C was Anisotropy = FA_free_ + (FA_bound_-FA_free_).[ExoiX]/(Kd + [ExoIX]), where FA_free_ is the FA of the unbound oligo, FA_bound_ is the FA of the fully bound oligo and [ExoIX] is the ExoIX concentration. The equation was fitted to the data by non-linear regression using GraphPad Prism.

### Preparation of ExoIX-Lys67Ala mutant

Site-directed mutagenesis was carried out on a bacteriophage M13 derivative carrying the *xni* gene as described previously (27,38), subcloned into pJONEX4 expression vector and expressed and purified as described for the wild-type protein (27).

### FEN activity of ExoIX

A flap oligonucleotide substrate (Supplementary Figure S2C) was prepared by annealing together two oligonucleotides each at 500 pM; 5′-fluorescein modified -dAAAACGCTGTCTCGCTGAAAGCGAGACAGCGAAAGACGC TCG T and 5′-dACGAGCGTCTTTA in 20 mM Tris (pH 8), 1 mM EDTA and 500 mM NaCl. The mixture was heated to 80°C for 5 min and then allowed to cool to ambient temperature over 60 min. Annealed products were stored at −20°C. Reactions were carried out using 50 pM annealed flap substrate in 25 mM potassium glycinate (pH 9.3), 100 mM KCl, 1 mM DTT, 0.5 mM EDTA, with or without 10 mM MgCl_2_.

### Electrostatic surface potential calculation

The electrostatic surface charge potential of ExoIX was calculated for a model in which unobserved side chains had been added in their most likely rotamers. The calculation was then carried out and visualized at ±5 *k*_B_T/*e* using the APBS plugin (39) for pymol (40).

## RESULTS

### Overall structure of ExoIX

**Figure 1.**
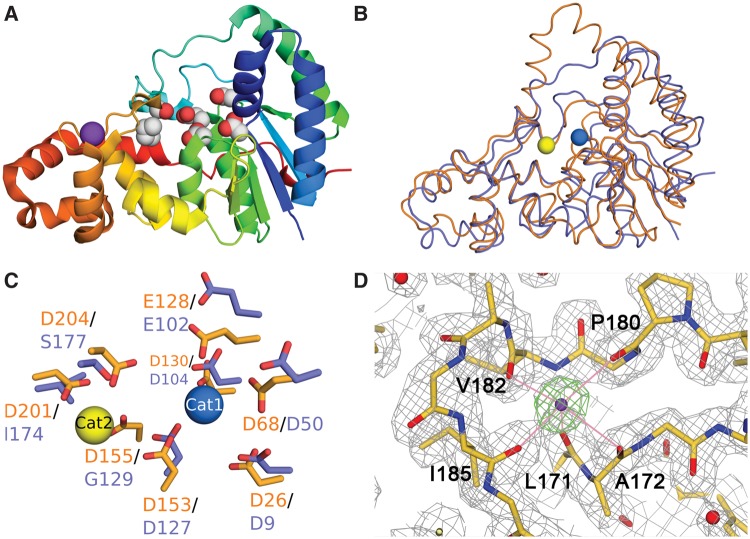
Overall structure of ExoIX. (**A**) Cartoon representing the fold of ExoIX, rainbow colored from blue at the N-terminal to red at the C-terminal. Side chains in the Cat1 and Cat2 sites are shown as white (carbon) and red (oxygen) spheres. The K^+^ ion is shown as a purple sphere. (**B**) Superposition of the structures of ExoIX (blue) and T5FEN (brown). The Cat1 and Cat2 sites of T5FEN are represented by blue and yellow spheres, respectively. (**C**) Detail of (B) showing superposition of the active sites of ExoIX (blue) and T5FEN (brown). The conserved residues in site Cat1 (right) and the substituted residues in site Cat2 (left) are indicated. (**D**) *2F_obs_-F_calc_* map showing the electron density in the region of the K^+^ ion (purple sphere) and its coordinating main chain carbonyls in the native ExoIX structure. Map contoured at 1 σ (grey); an *F_obs_-F_calc_* simulated annealing omit map for the K^+^ ion is contoured at 5 σ in green. Carbon, nitrogen and oxygen atoms shown in orange, blue and red, respectively.

The structure of ExoIX in the absence of DNA and of metal ions was determined by multiple isomorphous replacement in space group P1 at 2.0 Å. (see Supplementary Table S1). Later, a P2_1_ crystal form, also at 2.0 Å resolution, was obtained and solved by molecular replacement using one of the two essentially identical molecules in the P1 structure as a search model. Discussion of the native form will focus on the structure determined in P2_1_, unless otherwise stated, as it is more complete with more highly defined loop structures. The structure of ExoIX reveals a characteristic FEN fold with a central six-stranded mixed β-sheet surrounded by 10 α-helices, all linked by loops (Figure 1A and B).

Several other FEN structures have been reported, including T5FEN (3); the 5′ nuclease domain of Taq DNA polymerase I (6); the FEN-1 s of *Methanococcus jannashii* (41) and *Archaeoglobus fulgidus* (42); and the bacteriophage T4 RNAse H (4). Using the DALI server (43), these can be superimposed onto the structure of ExoIX with root mean square deviations (RMSDs) of 2.2 Å over 241 Cαs (Figure 1B); 3.0 Å over 212 Cαs; 3.8 Å over 198 Cαs; 4.1 Å over 211 Cαs; and 2.6 Å over 206 Cαs, respectively. The more distantly related hFEN1 (9) and hExo1 structures (10) can be superimposed with an RMSD of 4.0 Å over 219 amino acids and 3.7 Å over 217 amino acids, respectively, demonstrating the highly conserved nature of the FEN fold and active site.

As predicted by sequence comparisons ExoIX possesses only one of the two distinct metal-binding sites observed in other FENs (1). The structural superpositions show that the Cat1 site carboxylic acid residues Asp9, Asp50, Glu102, Asp104 and Asp127 superpose well on their respective counterparts Asp26, Asp68, Glu128, Asp130 and Asp153 in T5FEN (3) to form an essentially identical Cat1 site in ExoIX (Figure 1C). The Cat2 carboxylic acid side chains, however, are not present in ExoIX. The three aspartates that form this site in T5FEN (residues 155, 201 and 204) are replaced in ExoIX by Gly129, Ile174 and Ser177, respectively (Figure 1C). With the exception of the Ser177 hydroxyl and the side chain of Asp127 that lies at the boundary between Cat1 and Cat2, this produces a largely hydrophobic cavity, which would not be expected to bind metal ions in the same manner as the wider family of FENs.

### A novel potassium site in ExoIX

In both the P1 and P2_1_ ExoIX structures, a strong electron density peak was identified in the H3TH region of each protein chain. This density was directly octahedrally coordinated by the five main-chain carbonyl groups of Leu171, Ala172, Pro180, Val182 and Ile185, the sixth ligand being an ordered water molecule (Figure 1D). When the central peak was refined as a water molecule, it had a significantly lower B-factor than the surrounding protein, and therefore it was concluded that this must be a bound metal ion. Models were refined in parallel with K^+^, Na^+^, Mg^2+^, Ca^2+^ and Zn^2+^ cations each in turn occupying this site. The best agreement between the refined metal atom B-factor and that of the surrounding protein was given by K^+^ followed by Ca^2+^. The coordinating bonds for the atom were relatively long (average 2.92 Å), which, combined with the octahedral coordination geometry, was highly characteristic of a bound K^+^ ion, whereas Ca^2+^ would be expected to have significantly shorter bond lengths of ∼2.4 Å (44).

To confirm its identification as K^+^, and to investigate whether the metal ion was only present for stabilization of the ExoIX structure or for some other reason, the protein was purified and crystallized in the absence of potassium-containing buffers (see ‘Materials and Methods’ solution). The protein crystallized in space group C2 and the structure was solved by molecular replacement to 2.45 Å resolution (Supplementary Table S1). The ion-binding site was found to be unoccupied, which strongly supported its earlier identification as a K^+^ site. The K^+^-free structure is similar to the K^+^ bound structures, superposing on the P2_1_ native with an RMSD of 0.49 Å over all Cα atoms, suggesting that the K^+^ ion does not play a critical structural role in stabilizing the protein fold.

**Figure 2.**
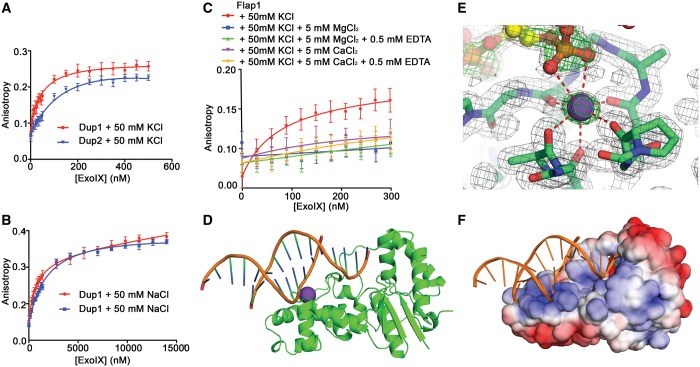
ExoIX binding to DNA. (**A, B**) Plots of FA against ExoIX concentration using the double-stranded oligonucleotides Dup1 (red) and Dup2 (blue) in the presence (A) and the absence (B) of KCl. (**C**) Plots of FA against ExoIX concentration using the Flap1 substrate under different conditions, colours as indicated in the Key. (**D**) Overview in cartoon representation of the complex between ExoIX (green) and 5ov6 DNA (orange backbone, blue/green bases.) (**E**) *2F_obs_-F_calc_* map (grey density) showing the K^+^ ion (purple sphere), its coordinating main-chain carbonyls and a DNA phosphate group in the ExoIX:5ov6 complex structure, contoured at 1σ. An *F_obs_-F_calc_* simulated annealing omit map in which the K^+^ and DNA are omitted from the refined model is shown as green density, contoured at 5 σ. The protein carbon atoms are shown in green, the DNA carbons in yellow. Nitrogen, oxygen and phosphorus atoms are colored blue, red and orange, respectively. (**F**) Electrostatic surface potential for ExoIX with DNA shown in cartoon representation. The K^+^ ion and DNA atoms were not included in the calculation, which was carried out and visualized at ±5 *k*_B_T/*e* using the APBS plugin (39) for pymol.

FA was used to investigate the effect of K^+^ on DNA binding to ExoIX. Initial experiments were performed using the oligomeric duplexes Dup1 and Dup2 (Supplementary Figure S2A and B). Plotting anisotropy against protein concentration (Figure 2A and B) for these substrates gave dissociation constants (*K*_d_) of 0.8 ± 0.1 µM and 2.0 ± 0.3 µM for Dup1 and Dup2, respectively, in the absence of K^+^. In the presence of 50 mM KCl, ExoIX showed a significant increase in affinity for the Dup1 and Dup2 duplexes with *K*_d_s of 45 ± 9 nM and 190 ± 60 nM, respectively. The influence of K^+^ on binding of a flap substrate to ExoIX was assessed using the same technique (Figure 2C). The substrate Flap1 (Supplementary Figure S2C) was labelled with fluorescein at the end of the 5′ flap. No DNA binding could be observed with µM concentrations of protein in the absence of K^+^ (data not shown). In contrast, in the presence of K^+^, DNA binding was detected with a *K*_d_ of 75 ± 30 nM. K^+^ therefore appears to greatly enhance the binding of ExoIX to the Flap1 DNA. Interestingly, in the presence of both K^+^ and Mg^2+^, no binding was observed, which is attributed to the cleavage of the fluorescently labelled flap from the DNA molecule.

A gel-based assay confirms that wild-type ExoIX is able to degrade the Flap1 substrate (Supplementary Figure S2F) in the presence of Mg^2+^. Mutation of the active site residue Lys67 to alanine greatly reduced this activity, but did not abolish it as observed in the equivalent mutation (K83A) in the homologous T5 FEN (13). Although we previously reported a lack of FEN activity in ExoIX (27) on a variety of different substrates, these results now provide biochemical evidence that ExoIX is able to cleave a double-flap substrate.

### Structure of the ExoIX:DNA complex

Co-crystallization with Flap1 was attempted, but instead crystals were obtained in which the Flap1 DNA had been processed into smaller fragments some of which then bound to the protein. The density for the backbone of double-stranded B-form DNA was clear, but the density for the bases was ambiguous, presumably corresponding to a sub-sequence of the original Flap1 averaged over a crystallographic 2-fold axis that relates the two strands. Two new DNA sequences for co-crystallization were designed to more ideally correspond to the oligonucleotide that could be observed in the ExoIX:Flap1 complex electron density map. These consisted of a 12-base or a 14-base palindromic sequence specifying eight base pairs and a four-base or a six-base 5′ overhang (Supplementary Figure S2D and E). These oligos (named 5ov4 and 5ov6, respectively), designed so their 2-fold symmetry would coincide with the crystallographic 2-fold axis, crystallized isomorphously with the original crystals of the ExoIX:Flap1 digestion product complex (Supplementary Table S1). The structures of the 5ov4 complex in the presence of Mg^2+^ and of Ca^2+^ were both determined at 1.5 Å resolution by molecular replacement using the ExoIX P2_1_ native structure as the search model. Scaling and structure solution statistics are shown in Supplementary Table S1.

Initial difference maps showed electron density for a short region of duplex DNA above the H3TH motif of ExoIX (Supplementary Figure S3A). This motif is involved in DNA binding in the FEN family of proteins (4,9,10) and also harbours the K^+^ site implicated in DNA binding in ExoIX as described earlier in the text. As it lay on the crystallographic 2-fold, a single strand of DNA was modelled in the asymmetric unit, the other strand being generated by crystallographic symmetry. Eight base pairs of duplex were observed approximating B-form DNA (Figure 2D). In the single-stranded overhang region, the bases remain stacked, and the backbone conformation continues to approximate to B form DNA. However, the chain becomes increasingly disordered, likely due to flexibility, as the 5′ end of the nucleotide approaches the active site region, and its density becomes poor ∼7 Å from the Cat1 site. The 5′-nt terminal interacts with arginine 16, a residue conserved in the eubacterial FEN domains.

Examination of the ExoIX:DNA interactions shows the central role played by the K^+^ ion, which ion-pairs with a phosphate group on the DNA backbone (Figure 2E). In addition, a number of hydrogen bonds are formed between the protein and nucleic acid. Most of the interactions are formed with one strand of the DNA, and nearly all of them are localized to the H3TH region of the protein where the K^+^ site is located, as shown schematically in Supplementary Figure S4. Six direct hydrogen bonds are formed between the protein and one of the DNA strands (the one modelled in the asymmetric unit). All are from the H3TH region of the protein, and two of these are provided by the main chain amide groups of Gly184 and Gly186 from the GhG motif, with the main chain carbonyl group of I185 (the central hydrophobic amino acid in the GhG motif) acting as one of the K^+^ ligands. Two further hydrogen bonds are formed by the side chains of T126 and R16 to a phosphate in the second strand of the DNA duplex (Supplementary Figure S4).

In addition to the specific hydrogen bonds described earlier in the text, the surface of ExoIX has an overall positive charge in this region (Figure 2F), which, together with the K^+^ ion, complements the negative charge of the DNA backbone.

**Figure 3.**
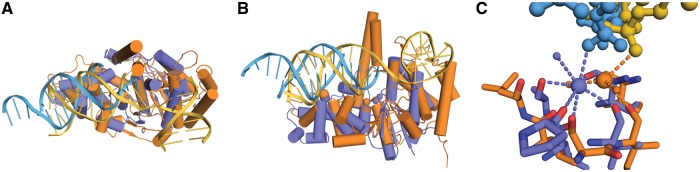
Comparisons of ExoIX with human FEN1. (**A**, **B**) Orthogonal views of the overall superposition of the ExoIX:5ov4:Mg^2+^ (blue and pale blue) structure with the hFEN1:DNA complex structure (orange and yellow), both shown in cartoon representation. The DNA moieties were not used to calculate the superposition. (**C**) Superposition of the H3TH motifs from ExoIX and hFEN1 coloured as in (A), with large spheres representing K^+^ ions and small spheres water molecules; protein oxygen and nitrogen molecules shown in red and dark blue.

The protein part of the Mg^2+^-bound ExoIX-DNA complex structure can be superimposed on the hFEN1 and hExo1 DNA complexes with RMSDs of 3.02 and 3.27 Å over 180 and 188 Cα positions, respectively. The core protein folds are well conserved, notably in the Cat1 site region and in the DNA-binding and K^+^-binding helix-turn-helix regions of hFEN1 (Figure 3) and hExo1, both of which are discussed later in the text. However, there are additional features present in the human enzymes not present in ExoIX. Notably, ExoIX lacks the additional C-terminal helices, which form the 3′ flap-binding pocket in hFEN1 and is therefore unlikely to bind the upstream region of a 5′ flap substrate in the same manner.

The key conserved YKXXR motif common to all bacterial and phage FENs is not present in the Taq polymerase structure (6). Superpositions of the structures of ExoIX and hFEN1 indicate that the arginine (Arg70) is equivalent to Arg100 in hFEN1; the side chain of the latter hydrogen bonds to the phosphate group after which cleavage takes place. Therefore, a similar role can be envisioned for Arg70 in ExoIX and the equivalent residues in all the bacterial and phage FENs.

### Mg^2+^ and Ca^2+^ binding in the presence of DNA

**Figure 4.**
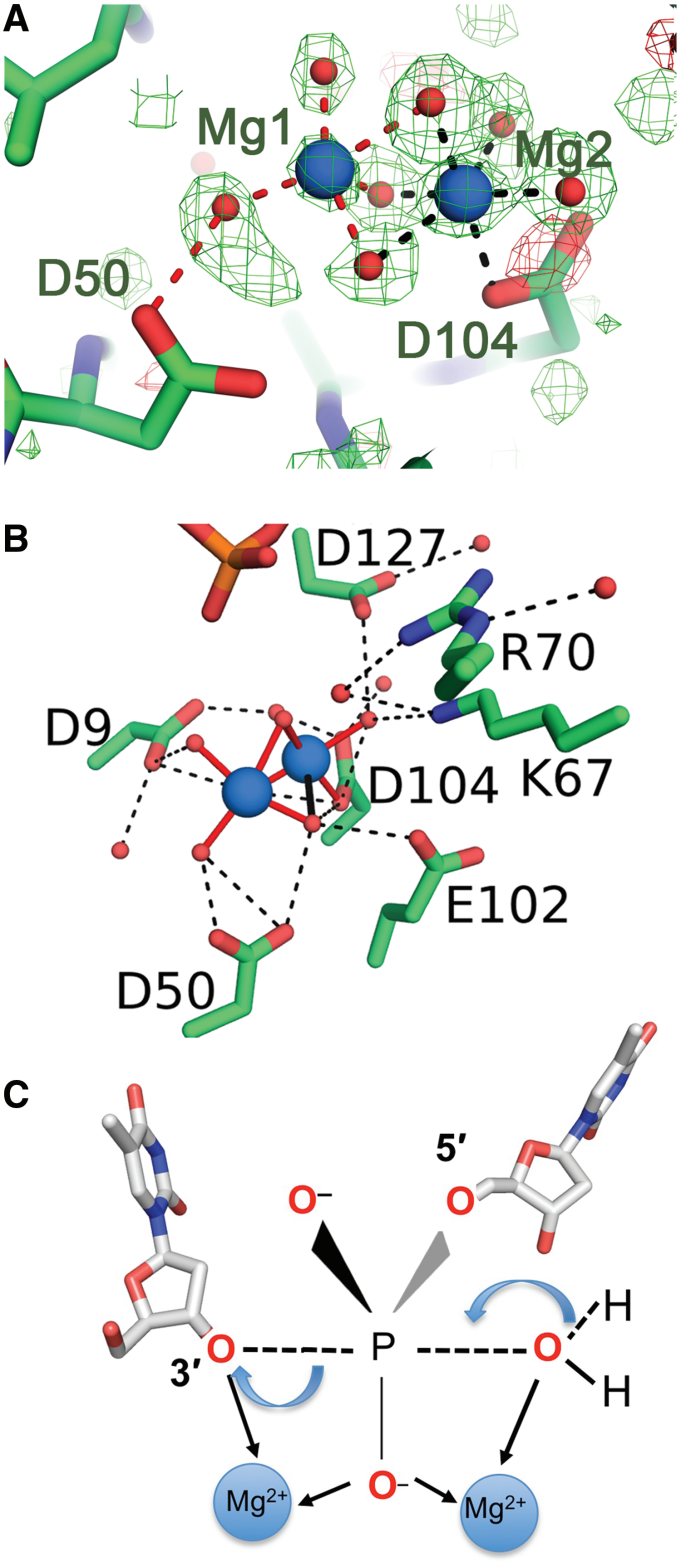
Mg^2+^ binding to the ExoIX:DNA complex in the Cat1 site. (**A)** *F_obs_-F_calc_* simulated annealing omit map, in which the Mg^2+^ ions and O atoms were omitted from the refinement, for the ExoIX:5ov4:Mg^2+^ complex showing the coordination of the two Mg^2+^ ions (blue spheres), contoured at 3 σ (green density positive, red negative), bonds associated with Mg1 shown as red, those with Mg2 as black dashed lines. (**B**) Coordination shells of the two Mg^2+^ ions (blue). Mg-O bonds are shown in solid red (<2.3 Å) or solid black (<2.5 Å); hydrogen bonds (<3.2 Å) to water or protein as black dashed lines. In both (A) and (B), Mg^2+^ ions are shown as large blue spheres, water/oxygen as small red spheres and protein/DNA carbon, phosphorus, nitrogen and oxygen atoms in green, orange, blue and red. (**C**) Schematic diagram showing a possible trigonal-bipyramidal transition state for hydrolysis of phosphate diester in ExoIX. In the proposed two-metal-ion mechanism, the divalent ions are usually separated by 3.5–4 Å, allowing one of them to lower the *pK*_a_ of a water molecule, thus allowing it to act as a nucleophile on the scissile phosphate. The second divalent metal ion stabilizes the trigonal-bipyramidal transition state that results from the said nucleophilic attack on the bound nucleic acid and assists departure of the leaving group.

In the ExoIX:DNA complex crystallized in the presence of Mg^2+^, two octahedrally coordinated metal ions were observed 2.5 Å apart, bridged by three probable hydroxyl groups (Figure 4A and B). One magnesium ion is octahedrally ligated by the OD1 atom of Asp104 and by five other oxygen ligands, three of which are shared with the second magnesium ion. This second magnesium has a further two oxygen ligands (Figure 4A and B), the sixth octahedral coordination position being unoccupied. The oxygen atoms around this pair of magnesium ions are hydrogen bonded to the side chains of Asp9, Asp50, Glu102, Asp104 and Asp127, which comprise the conserved Cat1 side chains (Figure 4B) and to Lys67 (equivalent to Lys83 in T5FEN and conserved in all prokaryotic FENs). Although the Cat2 site in ExoIX comprises a largely hydrophobic cavity, a cluster of four water molecules can be observed binding between aspartate and the serine side chains in the ExoIX:5ov4:Mg^2+^ complex structure.

ExoIX:5ov4-complex crystals were also grown in the presence of the inhibitory metal ion Ca^2+^. A single Ca^2+^ ion was ligated in a bi-dentate manner by Asp127 (equivalent to Asp171 in hExo1) and by four water molecules. Ca^2+^ binding has only been crystallographically observed in one other FEN:DNA complex, that of the hExo1 D173A inactive mutant (10) where the Ca^2+^ is coordinated by a different carboxylate side chain, hExo1 Asp152.

In both the ExoIX:5ov4:Mg^2+^ and ExoIX:5ov4:Ca^2+^ structures, there is evidence for partial displacement of K^+^ by Mg^2+^ and Ca^2+^, respectively. An additional electron density peak was observed adjacent to that of the K^+^ ion in both of these structures (Supplementary Figure S3D and E). Examination of the B-factors for the K^+^ ion suggested that it may not be present at full occupancy, and the new electron density peak was another metal ion. This was supported by the additional peak being more electron dense in the Ca^2+^ structure than in the Mg^2+^ structure, and the coordinating bond lengths being consistent with these metal ions, which are in the ranges 2.05–2.15 Å for Mg^2+^ and 2.35–2.45 Å for Ca^2+^(45).

Finally, it is of interest to compare the 5ov4:Mg^2+^ complex with the *apo* ExoIX enzyme. A superposition reveals few changes in protein structure as a result of DNA binding, with an RMSD of 0.84 Å over 244 Cα positions. There are some small local movements to contact the DNA. The largest change in structure is observed in the loop that links the helices of the H3TH motif. This moves ∼1 Å for Ser175 to H-bond with the DNA phosphate backbone. Other side chain movements that were observed include a 90° rotation of the Ser189 side chain and a 2.2 Å movement of Lys128, both of which H-bond to the phosphate backbone of the DNA. There are further side chain motions as a result of magnesium binding in the active site. Asp104 rotates to directly coordinate Mg2 (Figure 4A and B), whereas Glu102 also rotates such that its side chain is oriented towards the metal ions. This appears to be due to longer range charge–charge interactions with the metal, as no bridging water molecule was observed between the side chain and the metals, and the side chain oxygen were too far away to directly coordinate an Mg^2+^ ion. Other than these small changes, there are no large molecular motions that result from binding the 5ov4 oligonucleotide, though there may be further changes on binding a larger substrate not observed here.

## DISCUSSION

As described earlier in the text, the structure of ExoIX reveals a typical FEN fold (Figure 1A) and superimposes (43) well with other FENs, most closely with T5FEN (3) with an RMS deviation of 2.2 Å over 241 Cαs (Figure 1B). Like many FENs, ExoIX has a flexible loop (residues 70–74) above the active site rather than a helical arch but this does not form a hole large enough for a single-stranded DNA to thread through. Indeed, in superpositions with the recent hFEN1 and hExo1 complex structures (9,10), the upper part of the loop clearly blocks the hole formed by the helical gateway. A conformational change would therefore be necessary if ExoIX were to use a threading mechanism as proposed for other members of the superfamily (9,46).

Comparisons of the ExoIX:DNA complex structures with the ExoIX structures determined in the absence of DNA revealed that there are only minor conformational changes in the protein on binding of DNA to the H3TH region. However, some of the residues in the H3TH motif undergo small movements to hydrogen bond to the phosphate backbone of the DNA. This is similar to the hFEN1 DNA complexes (9) and that of T4 RNase H in complex with a pseudo-Y substrate (4) in which no large structural re-arrangements were observed in this region compared with the native structures (7,8).

It is also of interest to consider the possible mode of DNA flap binding in ExoIX. Comparisons of the ExoIX:DNA complex structures determined here with other FEN:DNA complexes show that the duplex binds in a position broadly similar to the downstream duplex regions in the hExo1 (10), hFEN1 (9) and T4 RNase H (4) DNA complex structures. Figures 3A–C show the overall superpositions, based on the protein components only, of the DNA complexes of ExoIX on hFEN1 and hExo1. Compared with the two human enzymes, the DNA double helix in ExoIX is rotated by ∼5° about an axis through the active site region so as to approach the H3TH motif more closely (Figure 3A and C). As a result, equivalent DNA phosphates that are distal to the protein:DNA interface differ in position by up to 8 Å. Despite this, however, the DNA strands in regions close to the protein, namely the complementary strand at the top of the H3TH motif and the substrate strand in the active site region, superpose well to within 3 Å of each other. This difference in the angle of the DNA relative to the protein appears to be the result of the different conformation that the H3TH loop adopts in ExoIX compared with the corresponding H2TH motif in the human enzymes, and to differences in the K^+^ site, both discussed in more detail later in the text. This region in ExoIX then appears to steer the DNA towards the active site. Nevertheless, the convergence of the superposed DNA strands in the active site region indicates that the 5′ flap of a substrate will follow a similar path through the active site of ExoIX to those in the human enzymes.

It is also of interest to consider the K^+^ site and its role in DNA binding. A K^+^ site analogous to that observed in both hFEN1 and in one of three deposited structures of hExo1 (9,10) and also in the HhH motif of POLB (47) is also present in ExoIX. This can be occupied by K^+^ both in the presence and absence of DNA in ExoIX and plays a key role in DNA binding as demonstrated by the significant increase in affinity of ExoIX for both duplex and flap DNA observed in the presence of K^+^ (Figure 2A–C). In hFEN1, K^+^ is also reported to be necessary for DNA binding, and a K^+^ ion is observed in the H3TH motif of all three DNA complex structures (9). However, in the three hExo1:DNA complex structures, one has K^+^ in the equivalent position, one has Ba^2+^ and in the third, no metal ion is observed at this position (10). Nevertheless, this suggests that this feature can be expected to be common if not ubiquitous in members of this enzyme family.

In the native ExoIX structure, the K^+^ site is approximately octahedrally coordinated by five main chain carbonyl groups together with a water molecule (Figure 1D), which is replaced by a phosphate group in the DNA complexes (Figure 2E, Supplementary Figure S4). Apart from the well-known coordination of K^+^ by eight main chain carbonyl groups in the potassium channel (48), sites that are so heavily coordinated by main chain carbonyls do not appear to have been widely described. The H3TH motif of ExoIX appears better adapted to binding K^+^ than any other metal, as it provides five of the seven coordinating bonds through its main chain carbonyl groups with bond distances suitable for K^+^ binding (49), the other two being supplied by two oxygens from the same DNA phosphate group in the ExoIX:DNA complexes (Figure 2E, Supplementary Figure S4).

Using only the helices of the H3TH/H2TH motifs, ExoIX superposes on hFEN1 and hExo1 with RMSDs of 1.12 and 1.08 Å, respectively, over 25 α carbon positions. This superposition reveals two clear differences in this region between ExoIX and the human enzymes (Figure 3C). First, hFEN1 and hExo1 do not satisfy the K^+^ ions’ requirements as completely as ExoIX. Instead of the five coordinating main chain carbonyls found in ExoIX, hExo1 coordinates K^+^ with just two main chain carbonyls and one serine Oγ, whereas hFEN1 only provides one carbonyl to directly coordinate the K^+^, other coordination in the human enzymes presumably being provided by water molecules. This results in a difference in the position of the ion and also explains why these sites are not occupied in the absence of DNA in other structures. Second, the conformation of the loop connecting the helices is also different. In hFEN1 and hExo1, the loops are similar to each other and fall away from the DNA towards the helical gateway (Figure 3A and B), whereas in ExoIX, the contrasting conformation of the connecting loop allows the additional interactions with the K^+^ ion observed in ExoIX.

Thus, the site in ExoIX appears more specific for K^+^ than the site in hFEN1 as the ion is buried more deeply and more highly coordinated by the protein in ExoIX (Figure 3C). This likely explains why this site was occupied in native ExoIX, which has not been observed previously in any other non-DNA-bound FEN structure. Nevertheless, the K^+^ ion can still be removed without any adverse effects on the ExoIX structure as shown by our K^+^ free structure. In addition, the structures of the ExoIX:5ov4 complexes solved in the presence of high concentrations of Mg^2+^ and Ca^2+^ show these metal ions can partially displace K^+^ from this site, binding in a different position ∼1.5 Å from the K^+^-binding position (Supplementary Figure S3D and E), presumably because their coordination geometries cannot be correctly satisfied by the main chain carbonyl groups of the H3TH motif. This displacement of K^+^ as a result of Ca^2+^ binding in a different but mutually exclusive position may explain the weakening of DNA binding to ExoIX in the presence of Ca^2+^ with K^+^ observed in the solution FA studies (Figure 2C). It can therefore be concluded that K^+^ is the likely metal to be used by ExoIX for DNA binding in the cell where K^+^ concentrations can be as high as 200 mM (50). It is interesting in this connection to point out that ExoIX is the first structure of one of the family of *xni*-encoded FENs, all of which lack the Cat2 site. This site is conserved in all bacterial DNA polymerase I-FEN domains, and in all archaeal and eukaryotic FENs. It has been suggested that Cat2 may aid in DNA binding in hFEN1 (12), whereas Grasby and coworkers (14) have shown that Cat2 is involved in substrate binding in T5FEN. It is therefore tempting to suggest that the highly developed K^+^ site in ExoIX, which we have shown is essential for DNA binding, may have made the Cat2 site evolutionarily redundant for binding DNA in the *xni* FENs, and it is likely to be conserved in all the *xni* FENs. In contrast, the hExo1 K^+^ site is occupied in only one of the three published DNA complex structures (10), indicating that it is not obligatory, although it is occupied in all three published hFEN1:DNA complex structures (9).

In the active site, the ExoIX di-magnesium site also has mechanistic implications for the whole family of FENs. phosphoryl transfer reactions underpin the crucial processes of DNA replication, transcription and translation. Many of these essential enzymes catalyzing Phosphoryl transfer reactions require divalent metal ions for activity. Some nucleases such as homing endonucleases (51) and Holliday junction resolvases (52) bind only one metal ion in their active sites, which is sufficient for hydrolysis of the phosphodiester backbone. However, there is evidence that a two-metal-ion mechanism operates in many enzymes including the 3′–5′ exonuclease of DNA polymerase I (15), RNAse H (53), the type II and IA topoisomerases (54) and most recently hFEN1 (9) and hExo1 (10). These latter two structures reveal pairs of metal ions in their Cat1 sites (Sm^3+^ in hFEN1 and Ba^2+^ and Mn^2+^ in hExo1) separated by ∼4 Å and coordinating a DNA phosphate group (9,10). In hFEN1, the Sm^3+^ ions in Cat1 are also directly ligated by four carboxyl groups (9). However, in ExoIX, only one of the carboxyls (Asp104) directly coordinates the Mg^2+^ ions, whereas the others interact via water or hydroxyl groups (Figure 4A). This was also observed for the single Mg^2+^ ions in the Cat1 sites of *Methanococcus jannaschii* FEN (41) and in bacteriophage T4 RNaseH in the absence of DNA (7). It is not clear whether this indicates differences in the ligation of Mg^2+^ as opposed to Sm^3+^ or that these represent changes in the coordination shells of the ions in Cat1 as the DNA phosphate approaches.

In the proposed two-metal-ion mechanism, analogous to that seen in the 3′–5′ exonuclease of Klenow (15), the divalent ions are usually separated by 3.5–4 Å and flank the central phosphate diester (Figure 4C). An activated water molecule bound to the Mg^2+^ on the 5′ side of the scissile phosphate loses a proton, allowing formation of an O-P bond with concomitant breaking of the P-0 3′ bond. The leaving 3′ oxyanion may be stabilized by the second Mg^2+^ ion leading to release of 5′-phosphate and 3′-hydroxyl terminated products. Despite a large body of evidence in support of the two-metal-ion mechanism, there remains some controversy in the literature (16), and it has been suggested that the second metal ion, rather than being a necessity, may modulate the rate of cleavage as has been reported for some restriction enzymes (55). However, this is the first report of a crystal structure of a FEN with DNA and the biologically relevant cation Mg^2+^. Thus, the data reported here provide hard physical evidence supporting the two-metal-mechanism as well as providing the highest resolution yet reported of a FEN enzyme with or without DNA.

The ExoIX:5ov4:Mg^2+^ complex presented here reveals the presence of two magnesium ions bound in the Cat1 site with a separation of 2.5 Å, but there is no bridging phosphate group as the DNA molecule is too far away. The side chains and local structure defining the Cat1 site of ExoIX are highly conserved in other FENs (Supplementary Figure S1A and Figure 1B), and thus two metal ions may also be accommodated in the Cat1 site of these enzymes in the presence of DNA. This suggests a two-metal-ion mechanism of reaction can be performed in the Cat1 site with a third metal in the Cat2 site possibly involved in DNA binding in agreement with the observations of Feng *et al*. (13) and Syson *et al*. (56). This would explain how T5FEN can still function as an endonuclease even when the Cat2 site is compromised by mutation (13) and also the activity that we have observed here for ExoIX, which lacks nearly all of the Cat2 active site side chains typical of other FENs.

As the sequence of ExoIX shares 52% homology (32% identity) with the FEN domain of DNA polymerase I, our structural data provide a useful starting point from which to model this region of the PolI enzyme. Although the structure of the Taq polymerase FEN domain has been reported (1TAQ), as discussed earlier in the text, several key regions of this model were disordered, and although conserved aspartate residues in the PolI FEN domain are missing in ExoIX, our structure does include key conserved residues such as tyrosine, lysine and arginine present in the loop above the active site as well as the entire Cat1 site.

In conclusion, our high-resolution crystal structure determinations of native *E. coli* ExoIX and of ExoIX:DNA complexes have revealed the architecture of the first member of a widely distributed novel subfamily of FENs. They have a similar fold to other FENs but possess only one of the two canonical catalytic metal-binding sites characteristic of the FEN family. The observation of a novel K^+^-binding site coordinated by five main chain carbonyl oxygens from the H3TH motif is a key factor in DNA binding, in a manner reminiscent of that observed in hFEN1 (9) and hExo1 (10). However, the extent of the ion’s direct coordination by the protein is more extensive in ExoIX than in these related proteins, with five main chain carbonyls directly interacting with the ion compared with a single carbonyl oxygen in hFEN1, and two (together with one serine Oγ) in hExo1. FA experiments showed that binding of both duplex DNA and a flap substrate to ExoIX was significantly stronger in the presence of K^+^ than in its absence. The ExoIX:DNA complex structures also revealed direct interactions between the DNA phosphate backbone and the K^+^ ion bound to the H3TH motif of ExoIX, confirming its key functional role.

The structures presented also reveal that a pair of oxo-bridged magnesium ions binds in the Cat1 site of ExoIX in the presence of DNA. Given the conservation of the Cat1 site residues across the FEN family, this is likely to be the case in other FENs. These high-resolution crystal structures therefore lend firm support to the validity of the two-metal-ion mechanism for this family of enzymes.

## ACCESSION NUMBERS

Atomic coordinates and structure factors for the P1 native, the P2_1_ native, the C2 K+-free native, and the ExoIX:Flap1, ExoIX:5ov4:Mg2+, ExoIX:5ov4:Ca2+ and the ExoIX:5ov6 complexes have been deposited in the Protein Data Bank with accession codes 3zd8, 3zd9, 3zdc, 3zda, 3zdb, 3zdc and 3zdd, respectively.

## SUPPLEMENTARY DATA

Supplementary Data are available at NAR Online.

## FUNDING

BBSRC [50/D16001 and B19466] and BBSRC studentships (to G.R.H. and M.G.R.H.) and University of Sheffield studentships (to C.A.G. and C.S.F.); Diamond Light Source for access to beamlines I03 and I02 (BAG numbers MX300 and MX1218) that contributed to the results presented here. Funding for open access charge: University Publication Fund.

*Conflict of interest statement.* None declared.

## Supplementary Material

Supplementary Data
