# Supplementary material for: The structure of Escherichia coli ExoIX—implications for DNA binding and catalysis in flap endonucleases
Source: Nucleic Acids Res. 2013 Jul 2;41(17):8357–67. doi: 10.1093/nar/gkt591 (PMC3783174; doi:10.1093/nar/gkt591)
Supplement: Supplementary Data [file supp_41_17_8357__index.html]

The structure of Escherichia coli ExoIX—implications for DNA binding and catalysis in flap endonucleases — The structure of Escherichia coli ExoIX—implications for DNA binding and catalysis in flap endonucleases — Supplementary Data 

# The structure of *Escherichia coli* ExoIX—implications for DNA binding and catalysis in flap endonucleases

## 

files

**Files in this Data Supplement:**

- Supplementary Data - pdf file
