# Supplementary material for: The structure of Escherichia coli ExoIX—implications for DNA binding and catalysis in flap endonucleases
Source: Nucleic Acids Res. 2013 Jul 2;41(17):8357–67. doi: 10.1093/nar/gkt591 (PMC3783174; doi:10.1093/nar/gkt591)
Supplement: Supplementary Data [file supp_gkt591_nar-01117-m-2013-File006.pdf]

SUPPORTING FIGURES AND TABLE

A

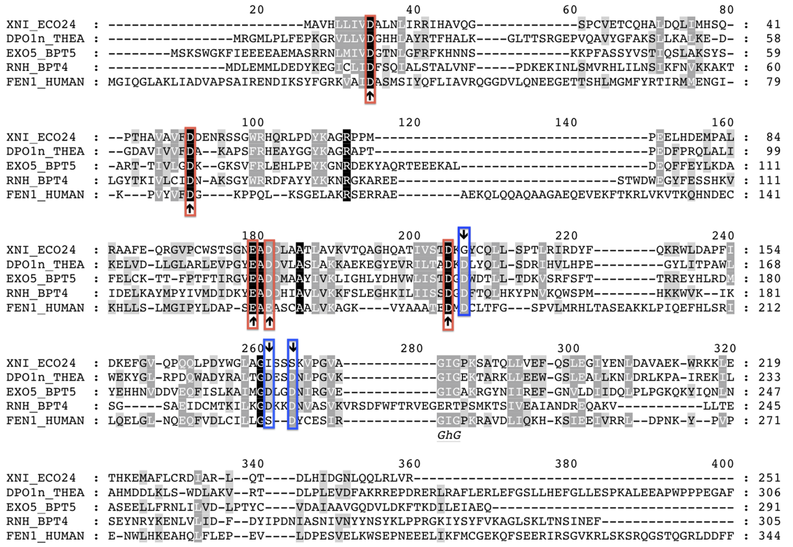

B

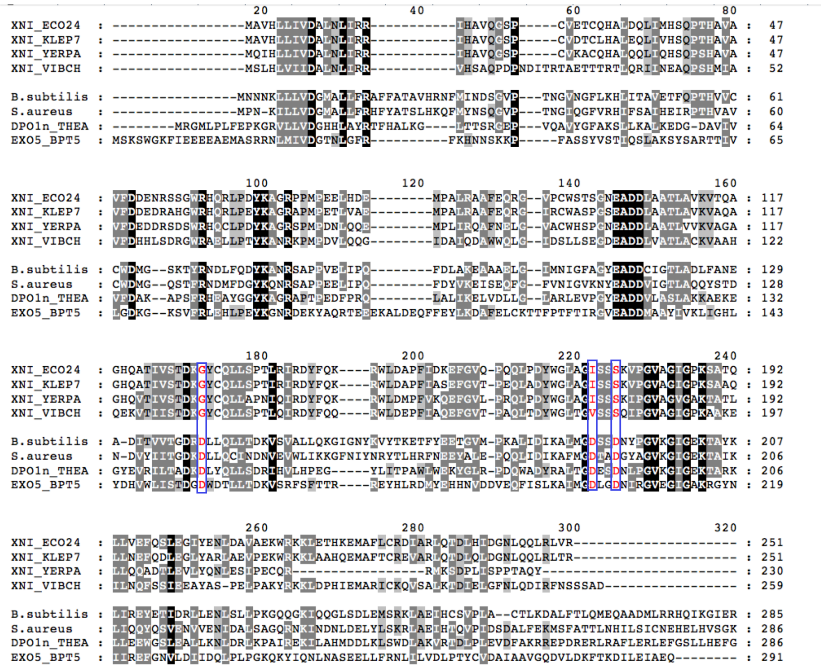

Figure S1. Sequence alignments of *E. coli* ExoIX against other FENs

(A) Alignment of ExoIX (XNI_ECO24) against the N-terminal domain of *Thermus aquaticus* DNA polymerase (DPO1n_THEA, N terminal domain of Uniprot DPO1_THEAQ), Bacteriophage T5 FEN (EXO5_BPT5), Bacteriophage T4 RNase H (RNH_BPT4) and human FEN-1 (FEN1_HUMAN). Black background indicates residues conserved across all these sequences, gray are partially conserved. Red boxes indicate Cat1 site residues, and blue boxes indicate the Cat2 site residues.

(B) Alignment of ExoIX (XNI_ECO24) against the Xni proteins from *Klebsiella pneumoniae* (XNI_KLEP7), *Yersinia pestis* (XNI_YERPA), *Vibrio cholerae* (XNI_VIBC), *B. subtilis*, *Staphylococcus aureus*, the N-terminal domain of *Thermus aquaticus* DNA polymerase (DPO1n_THEA) and bacteriophage T5 FEN (EXO5_BPT5). Black background indicates residues conserved across all these sequences, gray are partially conserved. The first four sequences are ExoIX-like Xni proteins as they lack the three Cat2 site aspartates (red letters in blue boxes).

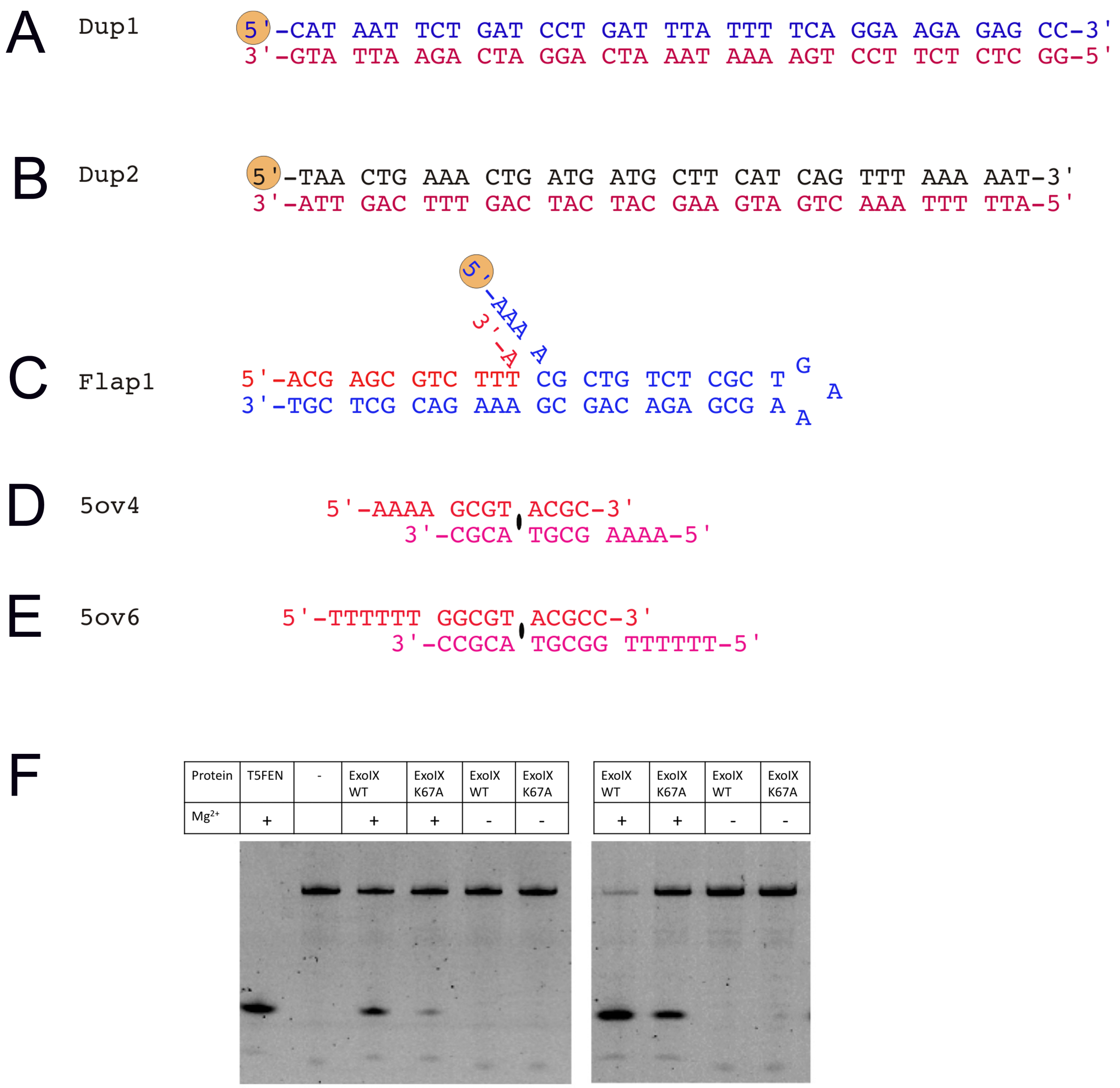

Figure S2. Oligonucleotide sequences and activity

(A,B) Sequences of Dup1 and Dup2 used in fluorescence anisotropy studies, orange circle indicates position of fluorescein label.

(C) Sequence of Flap1 used in fluorescence anisotropy studies and in initial co-crystallization study. Orange circle indicates position of fluorescein label.

(D,E) Sequences of palindromic oligonucelotides 5ov4 and 5ov6, respectively, used in the later co-crystallization studies. The position of the twofold symmetry axis is indicated by a filled black ellipse. **(F)** ExoIX displays nuclease activity on a flap substrate: fluorescently labelled Flap1 substrate (Figure S2C) was incubated with either T5 exonuclease (T5), wild type ExoIX (ExoIX WT) or ExoIX Lys67Ala mutant (ExoIX K67A) in the presence or absence of magnesium cofactor as indicated for 3hrs (left panel) or overnight (right panel). Substrate and product were analyzed by PAGE and visualized using a Fuji phosphorimager.

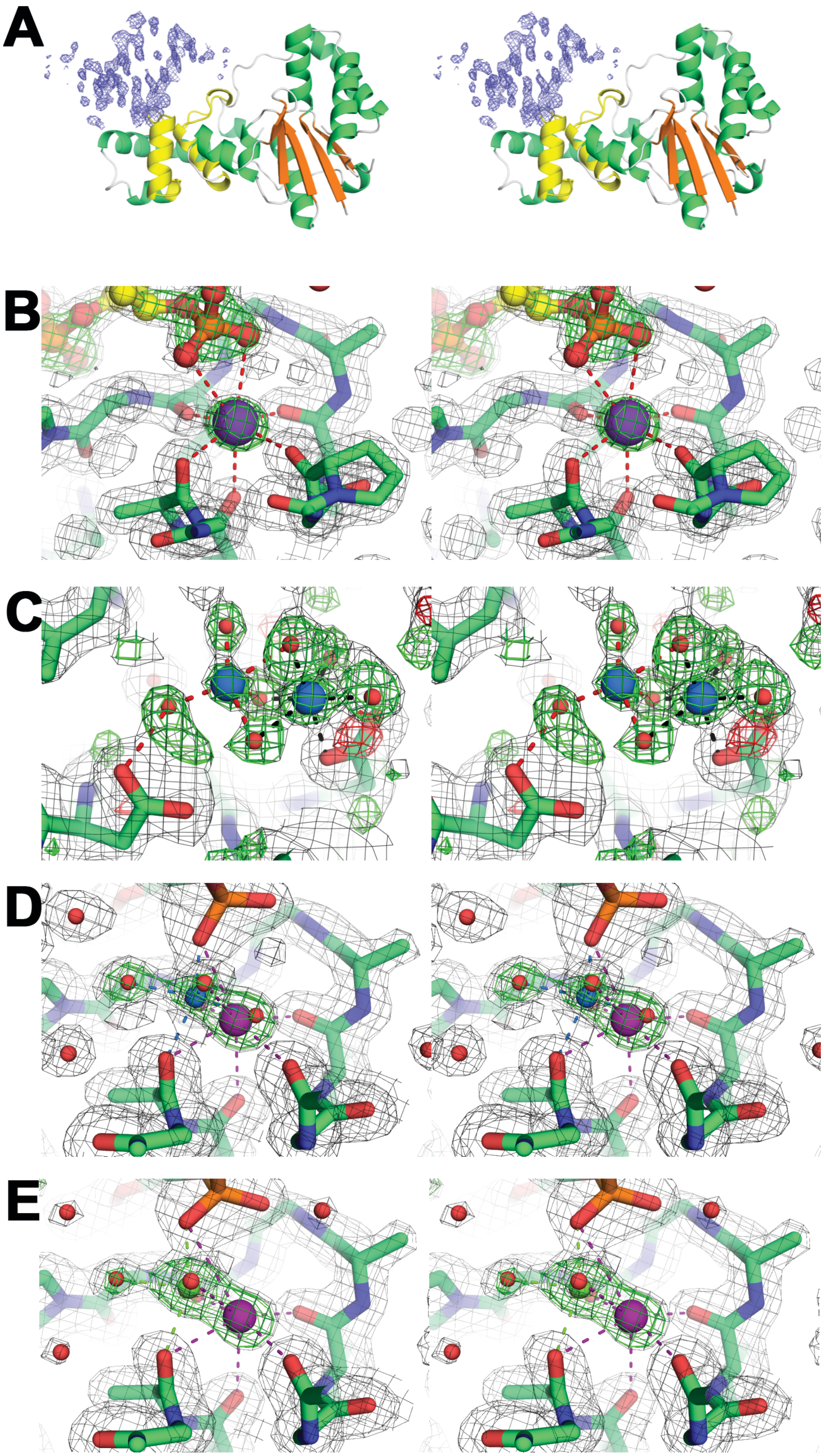

Figure S3. Stereodiagrams of electron density maps.

(A) View of the initial F_{obs}-F_{calc} difference map for the ExoIX:Flap1 complex showing density (blue) for the DNA duplex bound to the ExoIX molecule. Only the protein had been built into the model at this stage. The map is contoured at 2.5 σ. The ExoIX molecule is represented as a green cartoon with the H3tH motif highlighted in yellow and the beta sheet in orange.

(B) Stereo version of Figure 2E. *$2F_{obs}$-F_{calc}* map (grey, contoured 1 σ) at showing the electron density in the region of the K^+ ion (purple sphere) and its coordinating main chain carbonyls and DNA phosphate group in the native ExoIX:5ov6 complex structure. A *F_{obs}-F_{calc}* simulated annealing omit map (with K^+ and DNA excluded from the refinement) contoured at 5 σ is shown in green. The protein carbon atoms are shown in green, the DNA carbons in yellow. Nitrogen, oxygen and phosphorus atoms are colored blue, red and orange. Metal - ligand interactions are shown as dotted lines.

(C) Stereo version of Figure 4A. *F_{obs}-F_{calc}* simulated annealing omit map (green positive, red negative density, contoured at 3 σ; Mg^{2+} and nearby oxygens omitted from refinement) of the Cat1 site in the ExoIX:5ov4:Mg^{2+} complex showing the coordination of the two Mg^{2+} ions (blue spheres). An *$2F_{obs}$-F_{calc}* map (grey, contoured at 1 σ) is also shown. Other colors as Figure S3B.

(D) $2F_{obs}$-F_{calc} map for the K^+ site in the ExoIX:5ov4:Mg^{2+} complex (grey density, contoured at 1 σ) and F_{obs}-F_{calc} simulated annealing omit map in green (metal ions and nearby water molecules omitted, contoured at 5 σ) . The K^+ ion (0.4 occupancy, purple sphere) is partially displaced by the Mg^{2+} ion (0.6 occupancy, blue sphere).

(E) $2F_{obs}$-F_{calc} map (grey. contoured at 1 σ) and simulated annealing omit map (green, contoured at 5 σ) for the K^+ site for the ExoIX:5ov4:Ca^{2+} complex. The K^+ ion (0.4 occupancy, purple sphere) is partially displaced by the Ca^{2+} ion (0.6 occupancy, green sphere).

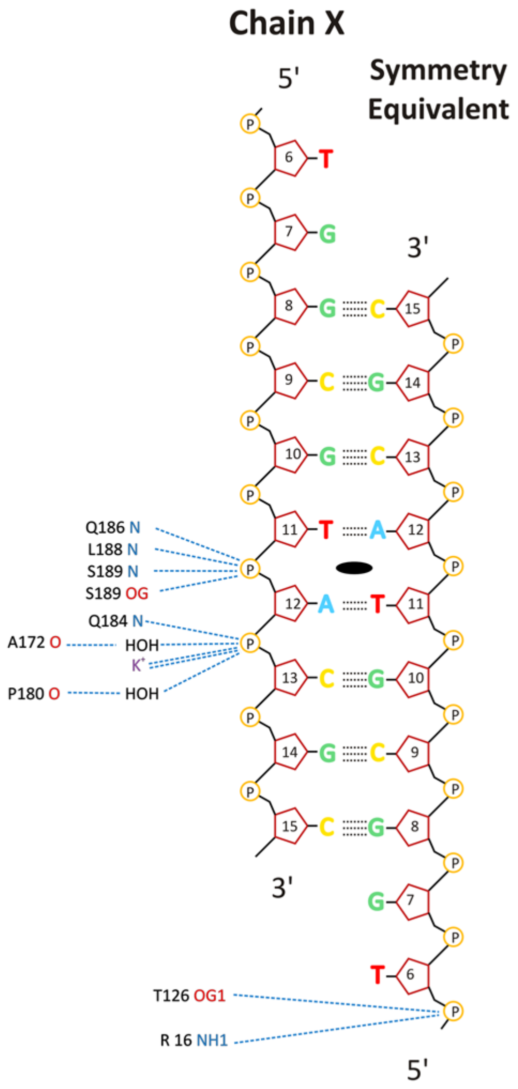

Figure S4. Schematic diagram of the interactions between ExoIX and the 5ov6 substrate.

Deoxyribose rings are represented as pentagons containing the residue number and the phosphodiester linkages as circles enclosing the letter P. Hydrogen bonds are shown as black (intra-DNA) or blue (protein-DNA) dotted lines. The crystallographic two-fold axis is represented by a filled black oval. The interactions between the reference ExoIX molecule and the DNA duplex are shown on the left, the identical ones from the other symmetry-related ExoIX molecule in the crystal are not shown.

.

Table S1. Data collection, MIR phasing and refinement statistics.

Dataset (pdb code)		Native (3zd8)	MIR Phasing HgSO$_4$	Au(CN)$_2$	Other Native Structures K$^+$ bound (3zd9)	K$^+$ Free (3zde)	Flap1 (3zda)	DNA Complex Structures 5ov4 + Mg^{2+} (3zdb)	5ov4 + Ca^{2+} (3zdc)	5ov6 (3zdd)
Space Group		P1	P1	P1	P2$_1$	C2	P2$_1$2$_1$2	P2$_1$2$_1$2	P2$_1$2$_1$2	P2$_1$2$_1$2
Cell Dimensions										
a (Å)	α (°)	43.4 110.4	43.4 110.6	43.8 110.9	53.5 90.0	128.5 90.0	66.2 90.0	66.2 90.0	66.2 90.0	66.4 90.0
b (Å)	β (°)	56.8 95.3	56.5 95.4	57.3 95.4	38.3 108.0	37.4 117.7	154.8 90.0	152.3 90.0	152.7 90.0	150.0 90.0
c (Å)	γ (°)	60.4 94.9	60.2 95.0	60.7 95.1	59.7 90.0	66.7 90.0	34.5 90.0	34.5 90.0	34.5 90.0	34.2 90.0
Resolution (Å) [b]		20.0 - 2.0 (2.05-2.00)	20.0 – 2.50 (2.64-2.50)	37.0 – 2.80 (2.95-2.80)	40.0 – 2.00 (2.05-2.00)	40.0 – 2.45 (2.58-2.45)	51.64 – 1.50 (1.54-1.50)	33.67 – 1.47 (1.51-1.47)	40.00 – 1.53 (1.57-1.53)	31.15 – 1.50 (1.54-1.50)
Beamline [a]		DL P.X 14.1	SHEF	SHEF	SHEF	SHEF	Diamond I03	Diamond I02	Diamond I02	Diamond I03
Detector		CCD	MAR 345	MAR 345	MAR345	MAR345	ADSC Q315 CCD	ADSC Q315 CCD	ADSC Q315 CCD	ADSC Q315 CCD
Wavelength (Å)		0.870	1.542	1.542	1.542	1.542	0.976	0.980	0.980	0.976
Observed/unique refls		82,213/34,557	23,621/15,496	24,719/12,138	41,565/14,512	18,686/9,692	571,962/57,905	246,258/60,541	223,277/53,386	307,026/54,849
R_{merge} (%) [b]		4.7 (30.8)	14.2 (56.4)	10.4 (42.5)	9.1 (36.9)	12.0 (40.9)	9.7 (47.7)	5.3 (23.8)	5.6 (42.2)	7.5 (51.6)
Completeness [b] (%)		92.3 (80.9)	84.6 (84.6)	90.5 (85.4)	89.0 (85.3)	92.4 (95.0)	99.9 (99.8)	99.9 (99.9)	99.3 (97.9)	98.1 (98.5)
I/σ [b]		8.8 (2.1)	4.9 (1.2)	6.2 (1.5)	11.4 (4.5)	3.8 (1.4)	22.5 (3.4)	15.2 (4.7)	13.7 (3.2)	12.6 (3.5)
[Heavy atom] (mM)		-	2	2	-	-	-	-	-	-
No. of sites		-	2	4	-	-	-	-	-	-
R_{iso} (%)		-	13.6	13.7	-	-	-	-	-	-
Phasing power 3.0Å		-	1.45	0.60	-	-	-	-	-	-
R_{cullis} 3.0Å		-	0.77	0.93	-	-	-	-	-	-
**Refinement Statistics**										
Number of reflections (working/free sets)		31,471/1,708	-	-	13,356/714	8,558/474	54,940/2,950	54,391/3,074	47,991/2,697	51,998/2,783
Number of atoms (protein/other)		3,726/249	-	-	1,947/100	1,895/17	1,915/206/257	1,918/245/211	1,901/245/170	1,957/206/229
R_{work}[c]/R_{free}[d]		0.160/0.227	-	-	0.159/0.246	0.170/0.275	0.211/0.235	0.201/0.231	0.205/0.229	0.215/0.254
RMS deviation from ideality:										
Bond lengths		0.016 Å	-	-	0.015 Å	0.008 Å	0.015 Å	0.013 Å	0.013 Å	0.018 Å
Bond angles		1.52 °	-	-	1.42 °	1.07 °	1.55°	1.44°	1.43°	1.62°
Dihedral angles		20.8 °	-	-	11.6 °	20.7 °	18.3°	18.2°	17.7°	13.9°
Bfactors of bonded atoms - overall		13.2 Å^2	-	-	16.0 Å^2	11.5 Å^2	6.6 Å^2	6.0 Å^2	5.3 Å^2	1.9 Å^2
main chain		6.7 Å^2	-	-	8.4 Å^2	10.7 Å^2	4.1 Å^2	3.7 Å^2	3.4 Å^2	1.4 Å^2
side chain		21.2 Å^2	-	-	25.3 Å^2	12.3 Å^2	9.0 Å^2	8.3 Å^2	7.1 Å^2	2.2 Å^2
DNA		-	-	-	-	-	36.7 Å^2	32.7 Å^2	18.6 Å^2	8.0 Å^2
Ramachandran plot. Proportion of residues in:										
allowed regions		98.3 %	-	-	98.8 %	97.5 %	99.2%	99.2%	99.6%	98.0%
additional allowed regions		1.7 %	-	-	1.2 %	2.1 %	0.8%	0.8%	0.4%	1.6%
disallowed regions		0.0 %	-	-	0.0 %	0.4 %	0.0%	0.0%	0.0%	0.4%
Average B-factors (main chain/side chain)		32.2/51.1 Å^2	-	-	22.8/43.6 Å^2	46.5/52.0 Å^2	25.4/31.5 Å^2	22.9/28.0 Å^2	25.0/30.8 Å^2	23.8/26.7 Å^2
Average B-factors of DNA		-	-	-	-	-	82.4 Å^2	65.7 Å^2	58.5 Å^2	65.7 Å^2
Number of waters/Average B-factor		247/47.5 Å^2	-	-	100/35.0 Å^2	17/ 42.0 Å^2	243/34.8 Å^2	203/31.1 Å^2	159/33.1 Å^2	220/35.6 Å^2
Number of metals/Average B-factor		2/26.4 Å^2	-	-	1/19.9 Å^2	-	2/32.0 Å^2	4/19.2 Å^2	3/25.1 Å^2	1/22.6 Å^2

[a] SHEF, on a MAR345 detector / Rigaku Micromax 007 rotating anode generator at University of Sheffield; DL, at CCLRC Daresbury Laboratories Synchrotron Radiation Source station P.X 14.1.

$R_{merge} = \Sigma|I - \langle I\rangle|/\Sigma I$, where I is the integrated intensity of a given reflection.

$R_{iso} = \Sigma|F_{PH} - F_P|/\Sigma F_P$, where F_{PH} and F_P are the derivative and native structure factor amplitudes.

Phasing power = <rms heavy atom structure factor>/<rms lack of closure> for acentric reflections (values for centric relections given in parentheses.)

R_{cullis} = <rms lack of closure>/<rms isomorphous difference> for acentric reflections. (values for centric relections given in parentheses.)

[b]values for the outermost resolution shell are given in parentheses.

[c]$R_{work} = \Sigma||F(obs)| - |F(calc)||/\Sigma|F(obs)|$ for the 95% of the reflection data used in refinement

[d]$R_{free} = \Sigma||F(obs)| - |F(calc)||/\Sigma|F(obs)|$ for the remaining 5 % of the reflection data excluded from the refineme
